# Application of the endogenous CRISPR-Cas type I-D system for genetic engineering in the thermoacidophilic archaeon *Sulfolobus acidocaldarius*

**DOI:** 10.3389/fmicb.2023.1254891

**Published:** 2023-10-02

**Authors:** Jan Bost, Alejandra Recalde, Bianca Waßmer, Alexander Wagner, Bettina Siebers, Sonja-Verena Albers

**Affiliations:** ^1^Molecular Biology of Archaea, Microbiology, Faculty of Biology, University of Freiburg, Freiburg, Germany; ^2^Molecular Enzyme Technology and Biochemistry (MEB), Environmental Microbiology and Biotechnology (EMB), Centre for Water and Environmental Research (CWE), Faculty of Chemistry, University of Duisburg-Essen, Essen, Germany

**Keywords:** archaea, genetic tools, deletion mutant, genetic engineering, type I-D CRISPR system, protospacer adjacent motif

## Abstract

CRISPR (clustered regularly interspaced short palindromic repeats)-Cas systems are widely distributed among bacteria and archaea. In this study, we demonstrate the successful utilization of the type I-D CRISPR-Cas system for genetic engineering in the thermoacidophilic archaeon *Sulfolobus acidocaldarius*. Given its extreme growth conditions characterized by a temperature of 75°C and pH 3, an uracil auxotrophic selection system was previously established, providing a basis for our investigations. We developed a novel plasmid specifically designed for genome editing, which incorporates a mini-CRISPR array that can be induced using xylose, resulting in targeted DNA cleavage. Additionally, we integrated a gene encoding the β-galactosidase of *Saccharolobus solfataricus* into the plasmid, enabling blue-white screening and facilitating the mutant screening process. Through the introduction of donor DNA containing genomic modifications into the plasmid, we successfully generated deletion mutants and point mutations in the genome of *S. acidocaldarius*. Exploiting the PAM (protospacer adjacent motif) dependence of type I systems, we experimentally confirmed the functionality of three different PAMs (CCA, GTA, and TCA) through a self-targeting assessment assay and the gene deletion of *upsE*. Our findings elucidate the application of the endogenous Type I-D CRISPR-Cas system for genetic engineering in *S. acidocaldarius*, thus expanding its genetic toolbox.

## 1. Introduction

The CRISPR-Cas system is an RNA-guided prokaryotic defense system to protect bacterial and archaeal cells from foreign DNA, such as virus invasion or conjugative plasmids (Barrangou et al., 2007; Brouns et al., 2008; Hale et al., 2009; Garneau et al., 2010; Marraffini and Sontheimer, 2010; Westra et al., 2012; Elmore et al., 2015). This self-defense mechanism consists of various steps (McGinn and Marraffini, 2019). First, the infected cell acquires a piece of foreign DNA and incorporates it into its own genome between specific clustered regularly interspaced short palindromic repeats (CRISPR). The integrated sequences are called spacers, which function as a memory from past survived infections (Fineran and Charpentier, 2012). Depending on the species, there are several of these clusters, which are accompanied by specific genes encoding for CRISPR-associated (Cas) proteins. CRISPR systems are divided into 2 classes, 6 types, and 33 subtypes and several variants, according to the properties of the Cas proteins (Makarova et al., 2015). Class 1 systems (type I, III, and IV) consist of a ribonucleoprotein (RNP) effector complex that is composed of several Cas proteins and bound crRNA (CRISPR RNA) during interference (Liu and Doudna, 2020). In comparison, class 2 systems (type II, V, and VI) only utilize one multidomain Cas protein, which interacts with crRNA for interference (Makarova et al., 2020; Nidhi et al., 2021). Approximately 47% of analyzed bacterial and archaeal genomes contain CRISPR systems, which, however, are much more prevalent in archaea (87%) than in bacteria (50%). Type I systems are the most dominant form of CRISPR systems, present in 64 and 60% of archaea and bacteria, respectively (Makarova et al., 2011, 2013). Type I and II systems interfere with invading DNA (Sinkunas et al., 2013), whereas type III systems, for example, interact with DNA and RNA in a transcriptional-dependent fashion (Samai et al., 2015).

In Sulfolobales, most CRISPR systems include type I-A, I-D, type III-B, and III-D (Vestergaard et al., 2014). Most research studies regarding CRISPR systems in Sulfolobales were performed in *Saccharolobus solfataricus* and *Sulfolobus islandicus*, showing the roles of different Cas proteins during CRISPR activity and the necessity of a protospacer adjacent motif (PAM) for type I systems (Peng et al., 2013), properties of protospacer and crRNA for interference (Manica et al., 2013; Mousaei et al., 2016), and the degradation properties of the type I-D system (Lin et al., 2020). For a more detailed insight into the different aspects of CRISPR-Cas systems in Sulfolobales, we refer to the reviews of the study mentioned in the reference (Garrett et al., 2011, 2015; Cannone et al., 2013; Manica and Schleper, 2013; Zhang and White, 2013; Zink et al., 2020).

After exploring various aspects of CRISPR-Cas systems in Sulfolobales, the endogenous CRISPR type I and III systems in *S. islandicus* were utilized for genome editing (Li et al., 2016). In this approach, the endogenous CRISPR-Cas system was primed using a specific spacer incorporated into a mini-CRISPR array integrated into a plasmid. By targeting a wild-type sequence, the CRISPR system induced DNA damage at the desired genomic site. Notably, the plasmid itself was not targeted due to the absence of a PAM sequence. The resulting DNA damage was subsequently repaired by cellular DNA repair mechanisms, thereby enabling the introduction of genetic modifications through homologous recombination with a provided repair fragment (Yang et al., 2020).

Furthermore, Schleper et al. demonstrated the potential of CRISPR systems in Sulfolobales by utilizing the type III system for RNA interference assays in *Sa. solfataricus*. Their studies highlighted the versatility and applicability of CRISPR systems in this context (Zebec et al., 2016; Zink et al., 2021).

**Figure 1:**
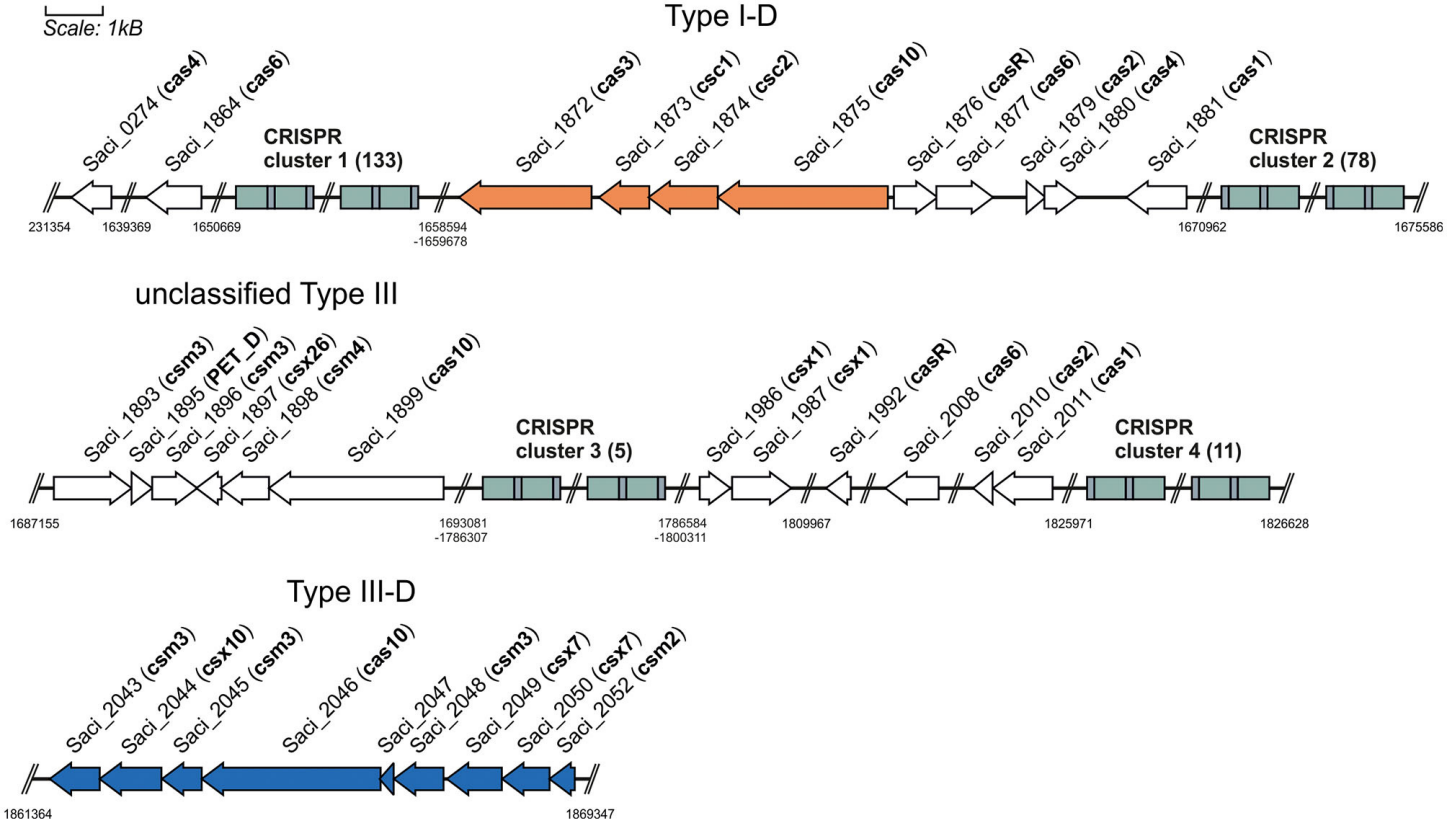
CRISPR loci in *S. acidocaldarius*. CRISPR type I-D and III-D systems are allocated on different loci of the genome. The Cas proteins (orange and blue, respectively) are associated with CRISPR clusters with different repeat sequences and different quantities of spacers. Furthermore, there are unclassified Type III system and other Cas proteins (white) that probably do not form a functional Cas cluster.

*S. acidocaldarius* exhibits CRISPR-Cas type I-D and III-D systems (Figure 1) (Makarova et al., 2020). The type I-D system is a unique type I system, as it does not have the Cas8 scaffold protein, but the Cas10 protein, which originates from a type III system, leads to the hypothesis of an evolutionary link between type I and III systems (Lin et al., 2020). The cluster contains the signature protein from the type I system, Cas3′ (helicase domain), without the endonuclease domain (Cas3″), as well as a Cas10-like large subunit, Cas10d from the type III system, together with Csc1 (Cas5) and the backbone Csc2 (Cas7) (Makarova et al., 2011). These Cas genes are associated with CRISPR clusters with different numbers of spacer sequences throughout the genome, with the type I-D genes associated with cluster 1 and cluster 2 (Figure 1).

To distinguish the foreign DNA from inherent DNA, type I and II systems need specific motifs that can be found next to the targeting sites (protospacer), called protospacer adjacent motif (PAM) (Mojica et al., 2009; Fischer et al., 2012). These PAMs differ in length and sequence between species and are located next to the protospacer at the 5′ end in type I systems and 3′ end in type II systems (Gleditzsch et al., 2019). These sequences can be discovered by analyzing the adjacent motifs of the protospacers of previous infections in the endogenous CRISPR clusters *via* bioinformatics tools. It is shown in *S. islandicus* LAL14/1 that the type I-D system is able to cleave dsDNA using the PAM GTN, similar to other type I systems, and also ssDNA by a ruler-like mechanism that is similar to type III systems without the need for a PAM (Lin et al., 2020). Previously, Lillestøl et al. proposed CCN, GTN, and TCN as PAMs for different Sulfolobales species (Lillestøl et al., 2009).

In this study, we used the endogenous type I CRISPR system of *S. acidocaldarius* to generate deletion mutants and introduce point mutations in previously characterized genes. Using this as proof of concept, we were able to expand the genetic toolbox of this thermoacidophilic model organism.

## 2. Materials and methods

### 2.1. Strains and growth conditions

For all experiments, *Sulfolobus acidocaldarius* MW001 was used as the background strain and aerobically grown in Brock standard medium (Brock et al., 1972) supplemented with 0.1% (w/v) N-Z-Amine and 0.2% (w/v) dextrin at pH of 3 and 75°C. For inducing protein expression from plasmids containing a xylose-inducible promoter, dextrin was replaced with 0.2% xylose. For complementation of the uracil auxotrophy, 20 μg/ml of uracil was added to the medium.

To cultivate *S. acidocaldarius* on a plate, Brock medium that was concentrated two times supplemented with 6 mM CaCl_2_, 20 mM MgCl_2_, 0.2% (w/v) N-Z-Amine, and 0.4% (w/v) dextrin was mixed in equal amount with freshly boiled 1.4% (w/v) Gelrite (Carl Roth, Karlsruhe, German). Cultures on plates were incubated for 5–7 days at 75°C in humidified containers. To remove plasmids from cells, they were streaked out on the second selection plates, which contained an additional 10 μg/ml uracil and 100 μg/ml 5-FOA.

### 2.2. Competent *S. acidocaldarius* MW001 cells

*S. acidocaldarius* strain MW001 was grown in 50 ml of Brock medium supplemented with NZ-Amine, dextrin, and uracil. Upon reaching an optical density (OD_600_) of 0.5–0.7, a portion of the culture was transferred to 50 ml of fresh medium and harvested at an optical density OD_600_ of 0.2–0.3. The culture was cooled down on ice and then centrifuged for 15 min at 4000 × *g* at 4°C and washed three times with 30 ml and one last time with 1 ml of ice-cold 20 mM sucrose. The cells were resuspended in 20 mM sucrose to reach a final theoretical optical density of 20 and divided into a portion of 50 μl and immediately used for transformation or stored at −80°C without freezing in liquid nitrogen.

### 2.3. Transformation of competent *S. acidocaldarius* MW001 cells

To prevent restriction by the *Sua*I restriction system, plasmids were methylated prior to their transformation into *S. acidocaldarius*. For this purpose, *E. coli* ER1821 cells, containing the additional plasmid pM.EsaBC4I (New England Biolabs, Frankfurt am Main, Germany), were transformed with the obtained plasmids. The methylated plasmids were, then, purified and electroporated into competent MW001 cells, using a Gene Pulser Xcell (Bio-Rad, München, Germany) with a constant time protocol at 1.5 kV, 25 μF, 600 Ω in 1-mm cuvettes. Cells were regenerated for 30 min at 75°C in 450 μl Brock medium without pH adjustment. To recover the transformants, 50 μl of cell suspension was inoculated in 5 ml of Brock medium supplemented with 0.2% (w/v) xylose and 0.1% (w/v) dextrin and incubated for 2 days at 75°C. From these cultures, 150 μl were plated per selection plate.

### 2.4. Construction of plasmids

To obtain a base vector for using the endogenous CRISPR-Cas system, an expression plasmid named pSVA13134 was designed. To that end, *lacI/lacZ* was cloned into the *Nco*I/*Not*I cloning site of pSVAxylFX-Stop (van der Kolk et al., 2020) using primers 11670/11671 for amplifying the plasmid and 11672/11673 for amplifying *lacI/lacZ* (Table 1). The primers contained overlapping regions at each end, which consisted of the cluster 1 repeat (GTAATAACGACAAGAAACTAAAAC) and *Sap*I restriction site. In between the repeats was the *lacI/lacZ* cluster. Cloning was performed using *in vivo* assembly resulting in pSVAxylFX-CRISPR. The *lacS* (*sso3019*) gene of *Sa. solfataricus*, encoding a β-galactosidase, was also added using *Nde*I and *Nhe*I with the primers 11642/11643 resulting in pSVA13133.

**Table 1:** Primer list.

**Primer**	**Sequence (5^′^-3^′^)**	**Purpose**
**Primers for the CRISPR base vector**		
11642	GACTGCTAGCCCGCGGCTAATTAATAATACTA	fwd to amplify P_mal_ *lacS* and terminator
11643	GATCCATATGCCGCAATCTAATGAAAATGAGA	rev to amplify P_mal_ *lacS* and terminator
11670	CCATGGTACGTATTATCTTATCATTC	fwd to linearize pSVAxylFX-Stop
11671	GCCCGCGGCTAATTAATAATAC	rev to linearize pSVAxylFX-Stop
11672	GAATGATAAGATAATACGTACCATGGGTAATAACGACAAGAAACTAAAACTGAAGAGCGCGCCCAATACG	fwd repeat cluster 1 and lacI
11673	GTATTATTAATTAGCCGCGGGCTAGCTCGAGGTCGACGTTTTAGTTTCTTGTCGTTATTACTGAAGAGCGACGTCTTAATGCGC	rev repeat cluster 1 and lacZ
12042	AGAAAGTGGTCCCTTACTCTAGTGCGTGTC	fwd to remove XhoI/ApaI
12049	CAAGTCTCACTATACCAAATGAG	rev to remove XhoI/ApaI
12050	AAATCTACCGTTGTCAATTTTA	fwd to introduce *Apa*I
12051	TTCAGTAGGGCCCGTGTGAAAGCGGCCG	rev to introduce *Apa*I
**Primers for US/DS repair fragments**		
12904	GTACATCCATATGAACATTTACGAGAATATTTATTACGCTAAGG	fwd US/DS for Δ*upsE*
12905	AGAATGGGCCCCTTAATCTATCCTTAAGCGAAACG	rev US/DS for Δ*upsE*
12918	GTACATCCATATGCGAGATTACTCCGTTATTGTTAG	fwd US/DS for *upsE* Walker A motif change
12919	AGAATGGGCCCAGTTCAGACTCCACATCTAC	rev US/DS for *upsE* Walker A motif change
12922	ATTGGGTCCAACGGGATCTGGAGCTACTACATTATTAAAC	fwd overlap US/DS *upsE* Walker A motif change K232A
12923	AAAGCGTTTAATAATGTAGTAGCTCCAGATCCCGTTGGAC	rev overlap US/DS *upsE* Walker A motif change K232A
**Primers for self-targeting assay**		
13606	TATGCTCTTCAAACAGAAAATATCTCAAGGAGGGCGAGGAAGTATGCGAAAGGTAAGAAGAGCAAT	protospacer cluster 1 spacer 1 ctr w/o PAM
13607	ATTGCTCTTCTTACCTTTCGCATACTTCCTCGCCCTCCTTGAGATATTTTCTGTTTGAAGAGCATA	protospacer cluster 1 spacer 1 ctr w/o PAM
13608	TATGCTCTTCAAACccaAGAAAATATCTCAAGGAGGGCGAGGAAGTATGCGAAAGGTAAGAAGAGCAAT	protospacer cluster 1 spacer 1 with CCA PAM
13609	ATTGCTCTTCTTACCTTTCGCATACTTCCTCGCCCTCCTTGAGATATTTTCTtggGTTTGAAGAGCATA	protospacer cluster 1 spacer 1 with CCA PAM
13610	TATGCTCTTCAAACtcaAGAAAATATCTCAAGGAGGGCGAGGAAGTATGCGAAAGGTAAGAAGAGCAAT	protospacer cluster 1 spacer 1 with TCA PAM
13611	ATTGCTCTTCTTACCTTTCGCATACTTCCTCGCCCTCCTTGAGATATTTTCTtgaGTTTGAAGAGCATA	protospacer cluster 1 spacer 1 with TCA PAM
13612	TATGCTCTTCAAACgtaAGAAAATATCTCAAGGAGGGCGAGGAAGTATGCGAAAGGTAAGAAGAGCAAT	protospacer cluster 1 spacer 1 with GTA PAM
13613	ATTGCTCTTCTTACCTTTCGCATACTTCCTCGCCCTCCTTGAGATATTTTCTtacGTTTGAAGAGCATA	protospacer cluster 1 spacer 1 with GTA PAM
**Primers for genetic manipulation**		
11554	TATGCTCTTCAAACTTAAAACCTCTGAACATTCTGGAAGTTATCAATTCCTGTAAGAAGAGCAAT	fwd spacer targeting *upsE*, CCA PAM
11555	ATTGCTCTTCTTACAGGAATTGATAACTTCCAGAATGTTCAGAGGTTTTAAGTTTGAAGAGCATA	rev spacer targeting *upsE*, CCA PAM
12900	TATGCTCTTCAAACACGGGATCTGGAAAAACTACATTATTAAACGCTTTACGTAAGAAGAGCAAT	fwd spacer targeting *upsE* Walker A motif change K232A, CCA PAM
12901	ATTGCTCTTCTTACGTAAAGCGTTTAATAATGTAGTTTTTCCAGATCCCGTGTTTGAAGAGCATA	rev spacer targeting *upsE* Walker A motif change K232A, CCA PAM
12912	TATGCTCTTCAAACATCCCGGTAAAGAGATTTCTTTAGATATAGTCGCTGCGTAAGAAGAGCAAT	fwd spacer targeting *upsE*, GTA PAM
12913	ATTGCTCTTCTTACGCAGCGACTATATCTAAAGAAATCTCTTTACCGGGATGTTTGAAGAGCATA	rev spacer targeting *upsE*, GTA PAM
12914	TATGCTCTTCAAACTTGCCCGAGGGTCATAGGGTAGCAGCGACTATATCTAGTAAGAAGAGCAAT	fwd spacer targeting *upsE*, TCA PAM
12915	ATTGCTCTTCTTACTAGATATAGTCGCTGCTACCCTATGACCCTCGGGCAAGTTTGAAGAGCATA	rev spacer targeting *upsE*, TCA PAM

Primers 12042/12049 were used to delete additional *Apa*I and *Xho*I sites and 12050/12051 to move an *Apa*I restriction site to another position, using T4 PNK cloning, to expand the usable multiple cloning site of the resulting plasmid pSVA13134.

Repair fragments for homologous recombination, containing genetic modifications, were then cloned into the *Apa*I/*Nde*I restriction sites using the primers, as shown in Table 1. For that, an upstream and a downstream fragment of *upsE* were amplified from genomic *S. acidocaldarius* DNA, and both PCR products ligated, using overlap extension PCR, resulting in pSVA13271 (for *upsE* KO) and pSVA13280 (Walker A mutation K232A) (Table 2). Spacers for targeted CRISPR activity were generated by annealing the forward and reverse primers at 98°C for 10 s followed by 50°C for 10 s. The primer contained *Sap*I restriction sites, parts of the cluster 1 repeat, and target sequence for the CRISPR system.

**Table 2:** Plasmid list.

**Plasmid**	**Backbone**	**Feature**	**Restriction enzyme**	**Primer**
**CRISPR backbone plasmids**				
pSVAxylFX-CRISPR	pSVAxylFX-Stop	*lacI/lacZ* flanked by CRISPR Cluster 1 repeats	in vivo assembly	11670/11671 + 1672/11673
pSVA13122	pSVAxylFX-CRISPR	P_mal_ and *lacS*	*Nde*I/*Nhe*I	11642/11643
pSVA13133	pSVA13122	pSVA13122 without *Apa*I and *Xho*I restriction sites	T4 PNK	12042/12049
pSVA13134	pSVA13133	pSVA13133 with new *Apa*I restriction sites in MCS	T4 PNK	12050/12051
**Self-targeting assay**				
pSVA6640	pSVA13134	**no PAM** control	FX (*Sap*I)	13606/13607
pSVA6642	pSVA13134	**CCA** target		13608/13609
pSVA6647	pSVA13134	**TCA** target		13610/13611
pSVA6648	pSVA13134	**GTA** target		13612/13613
**PAM testing in KO scenario**				
pSVA13271	pSVA13134	1kb repair fragment US/DS *saci_1494*	*Apa*I/*Nde*I	12904/12905
pSVA13272	pSVA13271	Δ*upsE*, **CCA** PAM	FX (*Sap*I)	11554/11555
pSVA13273	pSVA13271	Δ*upsE*, **GTA** PAM		12912/12913
pSVA13274	pSVA13271	Δ*upsE*, **TCA** PAM		12914/12915
**Point mutation**				
pSVA13280	pSVA13134	1kb *upsE* Walker A K232A (AAA->GCT) US/DS	*Apa*I/*Not*I	12918/12919 + 12922/12923
pSVA13281	pSVA13280	spacer *saci_1494* w/CCA and Cluster 1	FX (*Sap*I)	12900/12901

For the self-assessment protocol, the protospacer of the first spacer of CRISPR cluster 1 (AGAAAATATCTCAAGGAGGGCGAGGAAGTATGCGAAAG) was cloned into the *Sap*I restriction site using FX cloning with primers 13606/13607 for the non-PAM control and primers 13608-13613 for the different tested PAMs CCA, GTA, and TCA, respectively (Table 1).

### 2.5. Blue-white screening and colony PCR for genotype analysis

Potential candidates were first selected by blue-white screening, spraying X-Gal (25mg/ml in DMF) diluted in a 1:4 ratio with dextrin [20% (w/v)] on visible colonies. Afterward, the plates were incubated for up to 4 h until colonies turned blue. To verify the genotype of potential mutants, blue colonies were lysed for 2 min in 10 μl 0.2 M NaOH. To prevent DNA denaturation, 60 μl of 0.2 M Tris (pH 7.8) was added, as well as 60 μl of ddH_2_O added to dilute the sample. After vortexing, 1 μl was used as a template for a 20 μl PCR reaction using the Phusion polymerase. MW001 DNA was used as a wild-type control. For analysis of deletion mutants, plasmid DNA was used as a negative control to ensure that the signal was due to genetic alteration, not from plasmid amplification. After the analysis of gel electrophoresis, PCR products were sequenced (Eurofins Genomics Europe).

### 2.6. Ultraviolet aggregation assay

UV treatment was carried out following the protocol described by Fröls et al. (2008). In total, 10 ml of culture with an optical density (OD) of 0.2 to 0.3 was exposed to 75 J/m^2^ of UV light at 254 nm using a UV Crosslinker device (Spectroline, Westbury, NY). The cultures were, then, incubated at 75°C for 3 h. To determine the number of aggregated cells after UV exposure, the cell culture was diluted to OD 0.2, and 5 μl spotted onto a microscope slide coated with a thin layer of 1% (w/v) agarose in Brock minimal medium. After drying the cell suspension, a coverslip was added, and pictures were taken in three fields per sample under a phase contrast microscope. The number of free and aggregated cells (≥3) was counted using the ImageJ cell counter (NIH, Bethesda, MD).

## 3. Results and discussion

### 3.1. The CRISPR-Cas base vector for genetic manipulations

Wagner et al. established a genetic toolbox for *S. acidocaldarius* in 2012 based on a uracil auxotrophic strain MW001 in combination with a number of plasmids usable for the construction of deletion mutants, or mutants in which genes were either mutated genomically or tags were added to the gene of interest (Wagner et al., 2012). Moreover, using this system, genes were placed ectopically into the genome for genetic modifications (Wagner et al., 2012). To this end, plasmids are integrated into the genomic DNA after transformation *via* homologous recombination and can be excised after specific selection using 5-FOA and uracil, leading to alteration of the genomic DNA.

**Figure 2:**
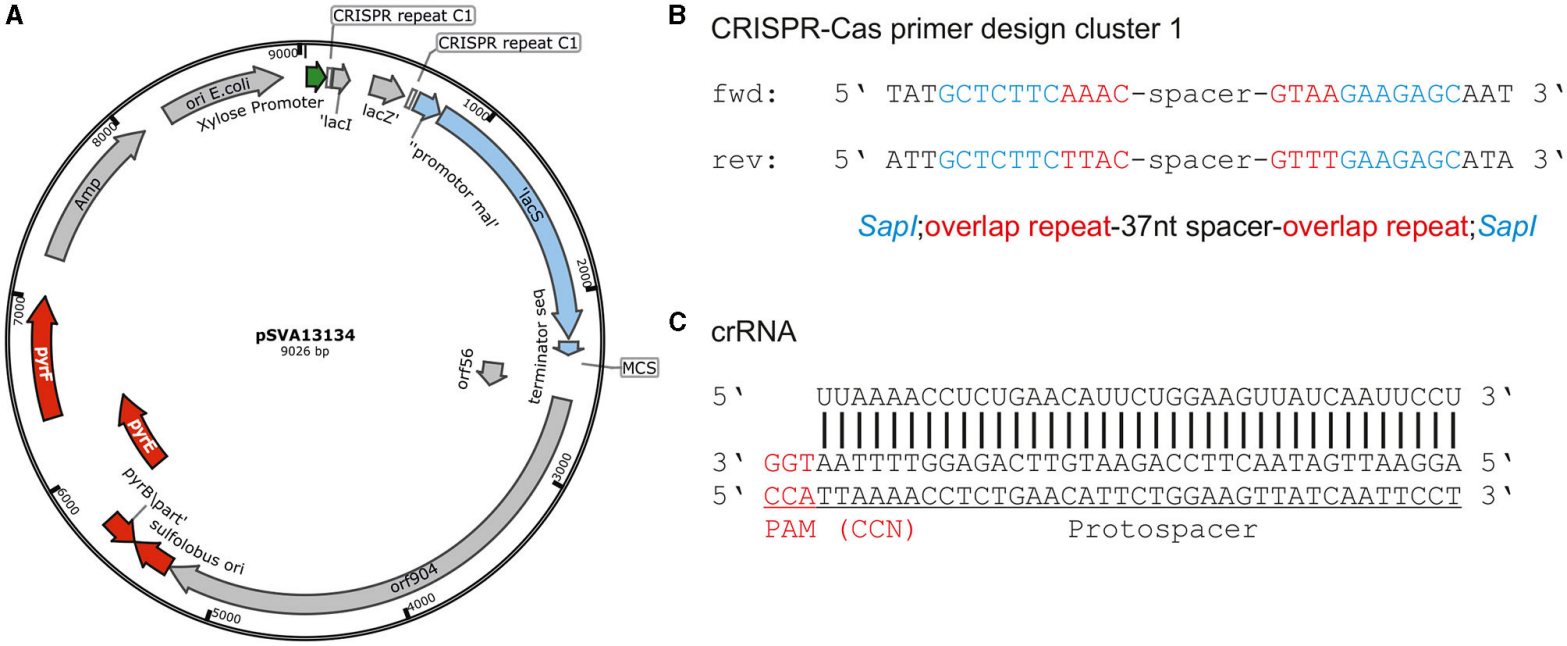
CRISPR base vector and CRISPR RNA design. **(A)** Plasmid map of base vector pSVA13134 containing xylose-inducible promoter P_xyl_/P_*saci*_1938_, repeats of CRISPR cluster 1 (C1), and *lacS*_*Sso*_ for blue-white screening in *S. acidocaldarius* under a maltose-inducible promoter. **(B)** Primer design for cloning of spacer sequence onto pSVA13134 using FX-cloning. Primer consists of *Sap*I restriction site, few nucleotides from overlap of cluster 1 repeats, and a specific spacer depending on the target sequence. The total length is ~65 nt. **(C)** Protospacer localization downstream of PAM (CCA) and CRISPR RNA binding to the target site for the deletion of *upsE*.

For the usage of the CRISPR-Cas system for genetic engineering, we designed plasmids based on the expression vector pSVAxylFX-CRISPR, which replicates in *S. acidocaldarius* does not integrate into the genome and is based on the plasmid pRN1 (Berkner et al., 2007). It derives from pSVAxylFX-Stop (van der Kolk et al., 2020) and contains 2 CRISPR repeats of cluster 1 (GTAATAACGACAAGAAACTAAAAC), which are downstream of a D-xylose-inducible promoter P_xyl_/P_*saci*_1938_. Additionally, *lacS*_*Sso*_ from *Sa. solfataricus* was integrated into pSVAxylFX-CRISPR, to allow for blue–white screening in *S. acidocaldarius* using X-gal and verify successful transformation. The final vector pSVA13134, which was the base for all plasmids used for genetic manipulations (Figure 2A), also contains a multiple cloning site that is suitable for inserting the repair fragment. The spacer/target sequence can be ordered as a primer pair and cloned into pSVA13134 by restriction with *Sap*I (Figure 2B). Spacer primers are designed by searching for a 37 nt protospacer sequence in the target area, which needs to be flanked by a PAM at the 5′ end (Figure 2C). Selection of positive *E. coli* clones is accomplished with blue–white screening because of the presence of the *lac*I*/lacZ* cassette in between the *SapI* restriction sites (Figure 2A) (Geertsma, 2013).

### 3.2. Introduction of recovery after transformation is important for CRISPR-based editing in *S. acidocaldarius*

**Figure 3:**
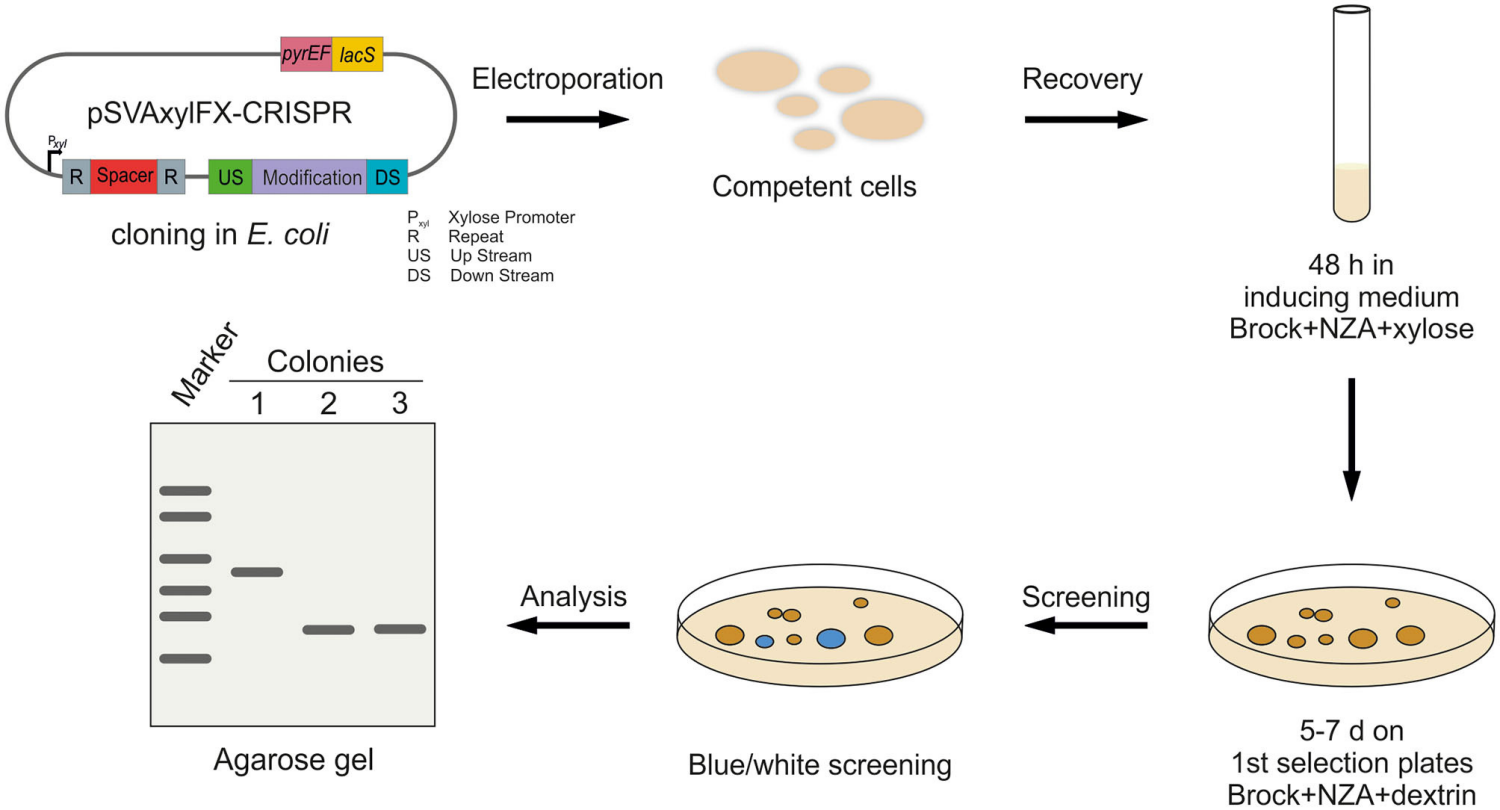
Workflow for CRISPR-based gene modification in *S. acidocaldarius*. After cloning of the CRISPR plasmids in *E. coli*, the methylated plasmid is transformed into competent *S. acidocaldarius* MW001 cells. Afterward, transformants are regenerated for 48 h in standard Brock medium supplemented with NZ-Amine and D-xylose for induction of the CRISPR system. Cells are, then, transferred to the first selection plates (NZ-Amine and dextrin) and incubated at 75°C for 5–7 days. After confirming plasmid presence using blue-white screening, blue colonies are further analyzed using PCR and agarose gel separation.

The CRISPR vectors used in this study are expression vectors, containing a *S. acidocaldarius* ORI. Therefore, a standard electroporation protocol for expression vectors was used, where transformed cells were plated on the first selection plates after 30 min of recovery at 75°C. However, we did not obtain any colonies with the CRISPR plasmids using this standard protocol. Therefore, we introduced an additional recovery step in the liquid medium after electroporation, which was similar to the lactose selection system in *Sa. solfataricus* PBL2025 (Albers and Driessen, 2008). Different recovery periods of 1, 2, and 3 d in Brock medium were tested, containing different carbon sources (D-xylose, sucrose, and dextrin). As the plasmid mini-CRISPR array is under the control of a D-xylose-inducible promoter P_xyl_/P_saci_1938_, induction of the CRISPR array is tested on plates, as well as in liquid medium for the 1–3 days of recovery step. Ultimately, positive genetically modified colonies only formed after 2 days of induction in Brock-NZ-Amine-D-xylose and plating on the first selection plates (Brock-NZ-Amine-dextrin) (Figure 3). No other combination yielded any positive colonies on plates.

In general, transformation in *S. acidocaldarius* always yields a certain amount of false positive colonies on the first selection plates, probably due to uracil cross-feeding from lysed cells. Therefore, transformants were diluted in a 1:100 ratio after transformation (50 μl transformants in 5 ml inducing medium for 2 days to prevent a high amount of background colonies, resulting in a ratio, that allowed for consistent colony formation).

### 3.3. The endogenous CRISPR-Cas type I-D system targets plasmid in self-targeting assessment assay

**Figure 4:**
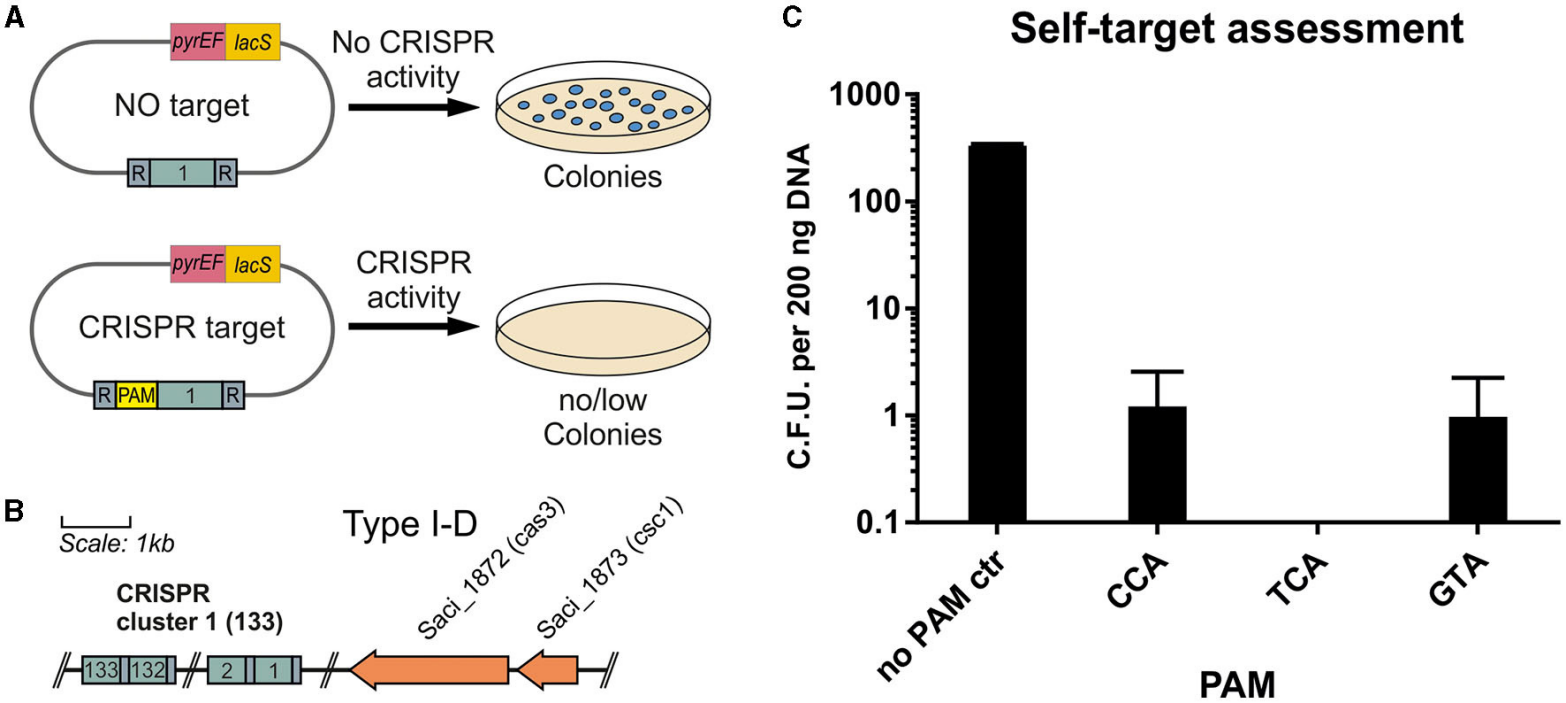
Self-targeting assessment of the endogenous CRISPR type I system. **(A)** Plasmids containing the corresponding target sequence of the first spacer from the CRISPR cluster 1 are used. **(B)** To verify an active system and functional cleavage capability, the target sequence is cloned onto plasmid pSVA13134 with and without a flanking PAM sequence. In the case of a non-functional PAM and CRISPR-Cas system, the plasmid is not cleaved, and colonies are able to form. In the case of a functional system, the plasmid is destroyed, and colonies are unable to form on the selective plates. **(C)** Colonies per 200 ng of plasmid DNA formed in the self-target assessment assay. As a control, the target is in between the native cluster 1 repeats leading to a no PAM control (AAC). Additionally, CCA, TCA, and GTA are used as PAMs accompanying the target at the 5′-end, leading to a possible recognition of the CRISPR-Cas complex. All 3 PAMs showed a high percentage of cleavage with 0 to 3 colonies forming per replicate. The average of three biological replicates is shown.

For *S. acidocaldarius, Sa. solfataricus*, and *S. islandicus*, several PAM sequences have been predicted bioinformatically by analyzing the targets of the protospacer sequences of CRISPR arrays from different Sulfolobales (Lillestøl et al., 2009). Three commonly found motifs were 5′-CCN-3′, 5′-GTN-3′, and 5′-TCN-3′. To verify the activity of the endogenous CRISPR type I-D system in *S. acidocaldarius*, a plasmid self-targeting assessment was performed. To that end, a plasmid containing a target sequence of the endogenous CRISPR-Cas system was transformed into *S. acidocaldarius* MW001. In the case of a functional CRISPR-Cas system and PAM sequence, the CRISPR-Cas RNP complex is able to cleave the plasmid, which also harbors a selection cassette, leading to the formation of fewer colonies compared with a non-target control (Figure 4A). Therefore, the protospacer sequence corresponding to the last acquired spacer of the CRISPR cluster 1 (AGAAAATATCTCAAGGAGGGCGAGGAAGTATGCGAAAG) was used as a target (Figure 4B). The sequence was flanked at the 5′-end by the PAMs CCA, GTA, or TCA in the CRISPR expression cassette, which was designed to mimic the sequence and arrangement of the native type I-D array in *S. acidocaldarius*. As a negative control, no PAM was inserted at the 5′-end of the target, which was just flanked by the native cluster 1 repeat sequence (AAC) (Figure 4A).

We showed that the presence of the target sequence for the first spacer of cluster 1 totally abolished the presence of the plasmid when using TCA as PAM. After blue-white screening to verify the presence of the plasmid, there was nearly full clearance for all tested PAMs with ~1 colony per 200 ng plasmid DNA for CCA and GTA PAMs (Figure 4C). The no PAM control yielded, on average, 303 colonies, showing a clearance effect for the used PAMs.

The self-target assessment indicated a functional CRISPR-type I-D system in *S. acidocaldarius*. The expression of endogenous Cas proteins under the native promoter system was sufficient to generate full clearance of transformed plasmids. Through this method, PAMs can easily be tested, as demonstrated previously, e.g., in *Pyrococcus furiosus* (Elmore et al., 2015). Notably, Lillestøl predicted CCN as a functional PAM for *Sa. solfataric*us and *S islandicus* as no valid PAM was predicted for *Sulfolobus acidocaldarius* (Lillestøl et al., 2009). Our results demonstrated that the three PAMs published by Lillestøl et al. have a similar impact on the clearing of targeted DNA.

### 3.4. The endogenous CRISPR-Cas type I-D system can be used for genetic engineering

#### 3.4.1. Deletion of *upsE*-proof viability of CRISPR-Cas as a genetic tool

**Figure 5:**
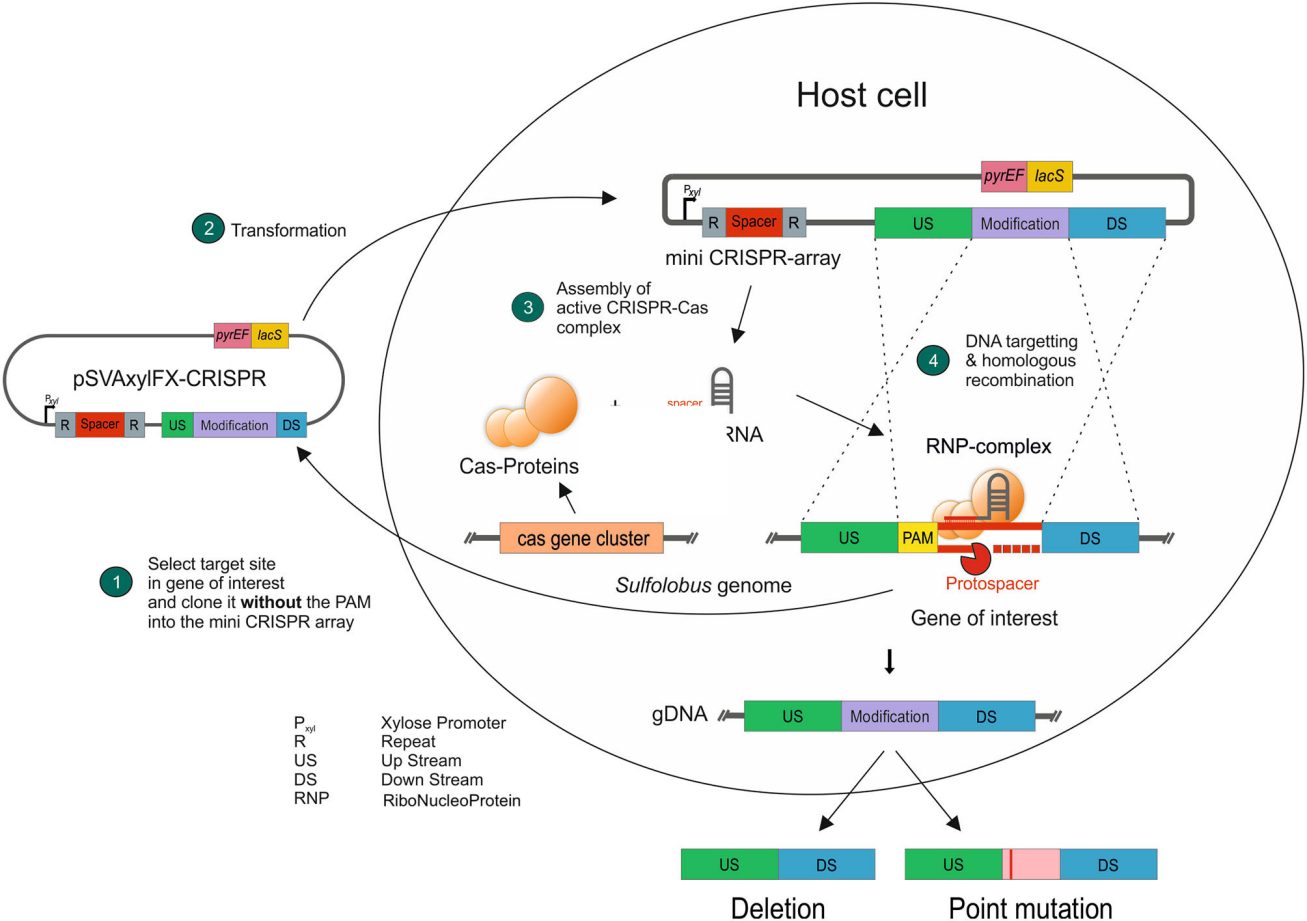
Concept of using the endogenous CRISPR-Cas system presented in this study. A target sequence next to a PAM is cloned into the CRISPR base vector, containing the genetic modification, without the PAM to avoid self-targeting (1). After transformation (2) and induction of the CRISPR array, the endogenous Cas proteins form a ribonucleoprotein complex (3), which scans the genomic DNA for the respective protospacer and then cleaves it. The genetically modified DNA sequence is implemented through DNA repair mechanisms *via* homologous recombination (4).

**Figure 6:**
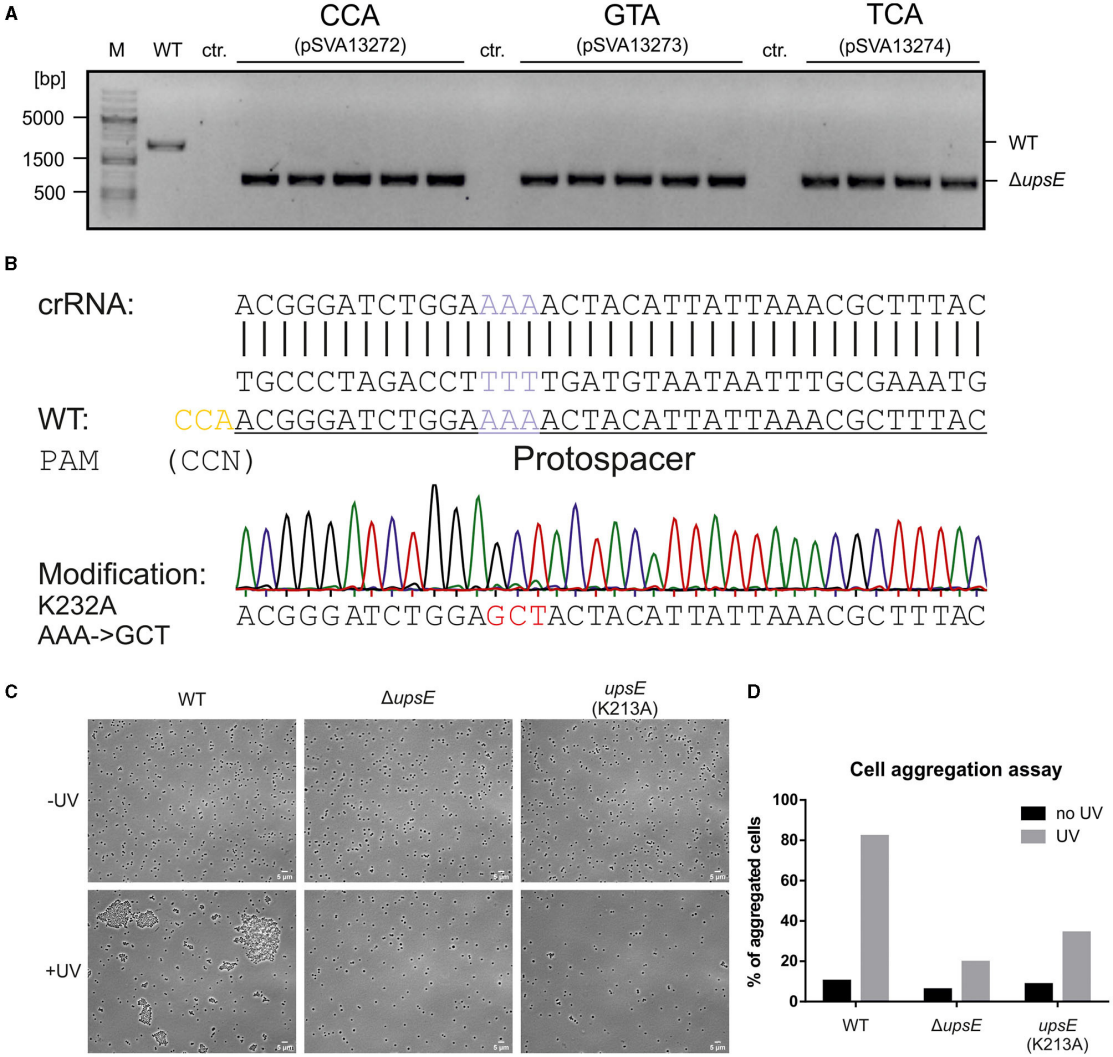
Constructing a deletion mutant and point mutations using the CRISPR plasmid. **(A)** Verification of Δ*upsE* deletion mutants using PCR. All three tested PAMs CCA, GTA, and TCA yielded positive signals for *upsE* deletion. MW001 (WT) DNA was used as a negative control. Control (ctr.) is the PCR reaction with the respective plasmid for the three PAMs alone, to ensure that the signal is due to genomic alteration and not due to the CRISPR plasmid. **(B)** The sequence of point mutation target site (purple) and the modification sequence (red) leading to an amino acid exchange from lysine to alanine at position 232 (K232A). The PAM CCA (yellow) is identified 12 bp upstream of the target triplet and the following 37 nt are used as a spacer for a targeted CRISPR cleavage. The mutation is verified using Sanger sequencing, shown here as a chromatograph. **(C)** Cell aggregation assay for the phenotypic analysis of the *upsE* deletion and point mutation mutants showing light microscopy phase contrast images of the control samples on the top row and the UV-treated cultures on the bottom row. **(D)** Analysis of cell aggregation assay. Shown are% of aggregated cells after UV treatment. The means correspond to three samples per biological triplicates of MW001 (WT), MW1301 (Δ*upsE*), and MW1304 (*upsE* K232A). Compared with the WT control, aggregation is impaired in both mutants.

After showing the activity of the CRISPR system and functional PAMs through the self-targeting assessment, we used *upsE* (*saci_1494*) as a target to test whether we can obtain a gene deletion using the system. *UpsE* encodes for the UV pili assembly ATPase (Fröls et al., 2008; Ajon et al., 2011; Wagner et al., 2012; van Wolferen et al., 2013). We chose it as a target as the successful deletion can be additionally verified *via* UV aggregation assays (Fröls et al., 2008). Therefore, we screened for PAM sequences in the sequence of *upsE* on the sense strand and used 37 nt downstream of it as a protospacer (Figure 5). The spacer sequence was cloned in between the CRISPR repeats of cluster 1 in the artificial CRISPR array on pSVA13271. This plasmid harbors a repair fragment for homologous recombination, which consists of the 500 bp upstream and 500 bp downstream of *upsE* and is derived from pSVA13134. We tested three different PAMs CCA, GTA, and TCA using the plasmids pSVA13272, pSVA13273, and pSVA13274, respectively. For transformation, 200 ng of plasmid was transformed, and the cells were incubated for 48 h in Brock medium with NZ-Amine and 0.2% (w/v) D-xylose. D-xylose induces the transcription of the CRISPR array on the plasmid, due to a D-xylose-inducible promoter, leading to the production of crRNA (CRISPR RNA), which then forms an RNP complex with the endogenous CRISPR-Cas proteins (Figure 5). After the formation of colonies on the first selection plates, initial screening of the presence of the plasmid was performed using X-Gal (5-bromo-4-chloro-indolyl-β-D-galactopyranoside) as the gene for the β galactosidase (*lacS)* is encoded on the plasmid. PCR analysis and subsequent sequencing confirmed that all blue colonies were indeed clones in which the *upsE* gene was deleted. There was no difference in either of the used PAMs CCA, GTA, and TCA (Figure 6A).

We were also able to cure the cells of the plasmid after verifying the genotype by putting the cells on the second selection plates containing 5-FOA, which was metabolized to a cytotoxic compound by the *pyrEF* gene, forcing the plasmid out of the cell (Wagner et al., 2012).

#### 3.4.2. Alteration of walker a motif of *upsE*

To further expand other possibilities for CRISPR-based genome editing, we tried to introduce a point mutation in the Walker A motif of *ups*E. In the case of successful genome alteration, the newly generated mutant should not be able to form aggregates upon UV induction similar to the deletion mutant. Therefore, we wanted to mutate a lysine residue at position 232 to an alanine (K232A) (Figure 6B), to abolish the ATPase function (delToro et al., 2016). For this, the target protospacer sequence needs to be spanning over the mutational site so that the crRNA only hybridizes with the WT sequence while abolishing targeting the mutation (Figure 6B). For *ups*E, a PAM CCA is localized 12 nt upstream of the target site, which is used. The modification was, then, put into a 1 kb repair fragment into the MCS of pSVA13134, containing the previously described point mutation. After following our established protocol, we were able to generate a Walker A mutant after the first transformation, named *S. acidocaldarius* MW1304, by only screening five clones, showing very high efficiency.

To verify the genetic edition, a UV aggregation assay was performed, showing impaired aggregation for both Δ*upsE* MW1301 and the Walker A mutant MW1304 (Figure 6C), as previously described (Fröls et al., 2008; Ajon et al., 2011; Wagner et al., 2012; van Wolferen et al., 2013). In contrast to the deletion mutant, there is still some aggregation of the Walker A mutant, but much less compared with the MW001 control, showing that the obtained mutants behave as expected (Figure 6D).

## 4. Conclusion

In this study, we have successfully demonstrated the utility of the endogenous type I CRISPR system in *S. acidocaldarius* as a versatile genetic tool for generating gene deletion mutants and introducing single codon changes within the *S. acidocaldarius* genome, similar to the previous findings in *S. islandicus* (Li et al., 2016). By testing various potential protospacer adjacent motifs (PAMs), we have expanded the range of available PAM sites, offering more options for targeted genetic modifications.

Although the cloning process for our method involves an additional step compared with the well-established “pop-in/pop-out” approach (Wagner et al., 2012), the identification of desired mutants can be confirmed on the first selection plate. Consequently, the acquisition of mutants in *S. acidocaldarius* is significantly accelerated compared with the previous methods. This expedited process is attributed not only to the increased yield of mutants, thereby reducing the screening period, but also to the fact that mutant screening is accomplished during the initial selection plates, ~10 days post-transformation. In contrast, the previous method necessitated screening after the second selection, which occurred ~16 days after transformation. It is worth noting that the second selection is still required to eliminate the plasmid from the cells, but this step is performed after confirming the genotype of the colony of interest.

These advancements in the application of the endogenous type I-D CRISPR system in *S. acidocaldarius* offer a significant improvement in both efficiency and speed, thus facilitating genetic manipulations and expanding the genetic engineering capabilities in this organism.

## Data availability statement

The original contributions presented in the study are included in the article/supplementary material, further inquiries can be directed to the corresponding author.

## Author contributions

JB: Investigation, Methodology, Project administration, Validation, Visualization, Writing—original draft, Writing—review and editing. AR: Investigation, Methodology, Project administration, Supervision, Writing—review and editing. BW: Investigation, Writing—review and editing. AW: Conceptualization, Investigation, Writing—review and editing. BS: Funding acquisition, Resources, Supervision, Writing—review and editing. S-VA: Conceptualization, Funding acquisition, Project administration, Resources, Supervision, Writing—review and editing.
